# The Effects of Red and Blue Lights on Circadian Variations in Cortisol, Alpha Amylase, and Melatonin

**DOI:** 10.1155/2010/829351

**Published:** 2010-06-24

**Authors:** Mariana G. Figueiro, Mark S. Rea

**Affiliations:** Lighting Research Center, Rensselaer Polytechnic Institute, 21 Union Street, 3rd Floor, Troy, New York, NY 12180, USA

## Abstract

The primary purpose of the present study was to expand our understanding of the impact of light exposures on the endocrine and autonomic systems as measured by acute cortisol, alpha amylase, and melatonin responses. We utilized exposures from narrowband long-wavelength (red) and from narrow-band short-wavelength (blue) lights to more precisely understand the role of the suprachiasmatic nuclei (SCN) in these responses. In a within-subjects experimental design, twelve subjects periodically received one-hour corneal exposures of 40 lux from the blue or from the red lights while continuously awake for 27 hours. Results showed-that, as expected, only the blue light reduced nocturnal melatonin. In contrast, both blue and red lights affected cortisol levels and, although less clear, alpha amylase levels as well. The present data bring into question whether the nonvisual pathway mediating nocturnal melatonin suppression is the same as that mediating other responses to light exhibited by the endocrine and the autonomic nervous systems.

## 1. Introduction

Circadian rhythms repeat every 24 hours (circa = approximately; die = day), reflecting the coupling of the self-oscillating endogenous master clock in the suprachiasmatic nuclei (SCN) with the natural 24-hour light-dark cycle. Perhaps the best known circadian rhythm is the synthesis pattern of the hormone melatonin by the pineal gland in the brain [1, 2]. The SCN closely regulate melatonin synthesis; melatonin concentrations, measured in plasma or saliva, are high during the night and low during the day. Melatonin is often called the “hormone of darkness” because its peak concentration levels are closely tied to the middle of the night in both nocturnal and diurnal animals. If light of sufficient irradiance, appropriate wavelengths and for sufficient duration is presented to the retina during the night, melatonin synthesis will be curtailed in a dose dependent manner [3].

Glucocorticoid hormones, of which cortisol is the most important one in humans, are steroid hormones produced by the adrenal cortex and participate in the body's homeostasis and stress responses [4–6]. Cortisol concentrations also follow a circadian rhythm [7] although one that is apparently more complex than the melatonin rhythm. Unlike the melatonin rhythm, human cortisol rhythms do not seem to be associated with day and night per se but seem to be more closely tied to the transition periods from dark to light and, to a lesser extent, from light to dark. In addition to its circadian rhythm exhibiting a predictable peak in the morning, cortisol levels typically elevate sharply in the morning, 30 minutes to an hour after awakening. The opposite appears true for nocturnal species, such as rats [8, 9], in that they exhibit a maximum corticosterone level and a sharp increase in its production in the evening with a broad nadir during the end of the night. Like humans then, the peak and spike in corticosterone amplitude in nocturnal animals are associated with the start of the daily activity period [10].

The glucocorticoid levels synthesized by the adrenal gland across the 24-hour day appear to be under the control of two distinct systems, one governed by the hypothalamic-pituitary-adrenal (HPA) axis [5] and one controlled by the autonomic nervous system through the adrenal medula [11, 12]. Adrenocorticotropic hormone (ACTH) release by the pituitary gland, the central part of the HPA axis, has traditionally been considered necessary for cortisol production and, indeed, under normal conditions ACTH levels follow a similar circadian pattern as cortisol [13, 14]. Although the average ACTH levels vary with circadian time, the momentary levels in a particular individual exhibit pulsatile spikes as, in fact, do cortisol levels [14]. These pulsatile spikes in ACTH occur throughout the 24-hour day, but interestingly cortisol spikes are much less frequent at night and do not appear to be systematically related to the sharp peak in cortisol associated with awakening [15]. Further evidence supports the conclusion that ACTH and glucocorticoid production can be decoupled [16]. Ablation of the SCN eliminates the ACTH circadian rhythm but not necessarily the production of corticosterone [11, 17]. Further, in rats, retinal stimulation by light during the night will modulate corticosterone production without affecting ACTH [11]. It has also been shown that this acute response to light at night is mediated by the SCN, possibly through the autonomic nervous system (sympathetic system) innervating the adrenal medulla [11, 18]. In one study, ablation of the SCN *or* the splanchnic nerve innervating the adrenal medulla eliminated the corticosterone response to light at night in rats [11]. Clearly then, the SCN seem to be tied to both the HPA and to the autonomic nervous systems as they affect glucocorticoid production. 

Salivary alpha amylase is an enzyme that has been used as a marker for the sympathetic nervous system response [19–22] and, like cortisol, has been shown to respond to psychosocial stress [19]. As with melatonin and cortisol, salivary alpha amylase production exhibits a regular circadian pattern [20]. During normal working schedules, salivary alpha amylase production roughly mirrors cortisol production across the 24-hour day [19]. In other words, under normal, steady-state conditions, high levels of cortisol tend to be associated with low levels of alpha amylase and vice versa. Interestingly, even the transient, awakening morning cortisol peak in humans is mirrored by a sharp, morning trough of alpha amylase concentration [23]. It is generally believed that the HPA and the sympathetic systems buffer one another as a negative feedback loop to minimize large swings in the organism's physiology when it is suddenly threatened [6], as exemplified by their approximately counterphased circadian rhythms and acute response to awakening in the morning. Only under significant and maintained environmental strain do the two systems respond similarly [20, 23], producing a positive feedback loop that incapacitates the organism (e.g., fainting) or moves it to action to eliminate the environmental threat [4]. When environmental strain persists, particularly without effective action by the organism, this positive feedback loop can have serious negative consequences to the organism's well-being [4]. 

Although many of the details regarding the role of the SCN in modulating the circadian, endocrine, and autonomic nervous systems' responses (melatonin, cortisol, and alpha amylase) remain unresolved, it appears very clear that the SCN affect the responses from these systems. Since light is a well-known stimulus for suppressing the synthesis of the hormone melatonin at night, it was considered important for the present study to also better understand how light might affect cortisol, another endocrine hormone, and alpha amylase, a marker of the sympathetic system. For example, previous studies have shown that exposure to high levels of polychromatic (white) light (800 lux [lx] at the cornea) in the morning, but not in the evening, increased cortisol levels in humans [24, 25]. Studies have also shown that morning light can increase heart rate, suggesting an impact of light on the autonomic nervous system [26].

The general purposes of the present study then were, first, to obtain simultaneous baseline measurements of three biomarkers, melatonin, cortisol, and alpha amylase, in the absence of light, to more closely examine the relationships between these three circadian rhythms during sleep restriction and in that context to, second, expand our understanding of the impact of acute light exposures on these biomarkers during the day and during the night. With regard to the second purpose, we utilized two lighting conditions, one-hour exposures of 40 lx each from a narrowband, short-wavelength (blue) and from a narrowband, long-wavelength (red) light, to more precisely understand the involvement of the SCN in the endocrine and autonomic nervous systems' responses to light during the day and during the night.

## 2. Materials and Methods

The present study was conducted in accordance with the Declaration of Helsinki (1964) and was approved by Rensselaer Polytechnic Institute's Institute Review Board (IRB).

### 2.1. Subject Selection

Twelve of sixteen subjects recruited from Rensselaer Polytechnic Institute in Troy, N.Y completed the repeated-measures study, and their data are reported here. Eight male subjects (ages = 20–40 years) and four female subjects (ages = 19–53 years) completed the study. Although the subject pool represents a wide age range and an unbalanced gender distribution, there is no reason to believe these factors confound the results because all subjects saw every condition in the within-subjects experimental design. Furthermore, there is little evidence that either age (for the range in our subject pool) or gender are systematically related to the amplitude or phase of melatonin, cortisol, or alpha amylase synthesis [22, 27, 28]. Subjects were screened for major health problems (heart disease, diabetes, asthma, and high blood pressure) by a research nurse via a phone interview and, except for two women taking oral contraceptives, for pharmaceuticals (including adrenergic agonists and antagonists). Subjects were also not allowed to take any nonsteroid anti-inflammatory medication starting 72 hours prior to the study. All subjects were nonsmokers and were asked to not consume alcohol or caffeine starting 12 hours prior to the start of the experiment. Subjects were asked to keep a regular schedule on the week of the experiment and report if there were any deviation from their normal schedule. If so, the subject was rescheduled to participate in the experiment on another Friday evening. None of the subjects had traveled more than three time zones in the month prior to the study. A few days prior to the experiment, subjects were asked to fill out a consent form and a Munich Chronotype Questionnaire (MCTQ) for the experiment [29]. The MCTQ data were used as assurance that subjects were not extremely early or extremely late chronotypes. All subjects reported having a chronotype between “moderately early” and “moderately late.” Moreover, all subjects reported having bed times no later than 23:00 and wake up times no later than 08:00 on weekdays and bed times no later than 00:00 and wake-up times no later than 09:00 on free days. The regular schedules increased the likelihood that all subjects were normal and homogenous with respect to circadian phase.

### 2.2. Lighting Conditions

Subjects were individually exposed for one hour to two experimental lighting conditions, two spectra (blue, B and red, R), both at one level (40 lx). For the third lighting condition, serving as the control condition, subjects sat quietly in a dim room (<3 lux at the eye and referred hereafter as dark, D). For the B and R lighting conditions subjects were individually positioned in a chin rest in front of a 0.6 × 0.6 × 0.6 m light box; each box was fitted with either an array of red or of blue light-emitting diodes (LEDs). The LED arrays (LXHL-MB1D (Lumileds, Luxeon I, blue [nominal 470 nm], Lambertian, 1 W, 350 mA and LXHL-MD1D (Lumileds, Luxeon I, red [nominal 625 nm], Lambertian, 1 W, 350 mA.)) were located behind the front box apertures to be outside the subject's direct view, thereby creating a uniform, nonglaring distribution of light within the box. The spectral emissions of the blue LEDs peaked at 470 nm with a full width at half maximum (FWHM) of 25 nm. Light from the red LEDs peaked at 625 nm with a FWHM of 25 nm. Before the experiment, each of the light boxes was calibrated using a Gigahertz illuminance photometer to provide the prescribed corneal illuminance levels when subjects were positioned in the chinrest. The spectral radiances of the red and blue lighting conditions were measured prior to the experiment with a calibrated spectroradiometer (PhotoResearch, model PR705a) and diffuse white reflectance standard (Labsphere, model SR 099); these were used to calibrate the Gigahertz illuminance readings during the experiment. Two boxes provided blue light (40 *μ*W/cm^2^ at 40 lx), and two emitted red light (19 *μ*W/cm^2^ at 40 lx) in the plane of the subjects' corneas; light levels could be adjusted with an electronic dimmer to reach the prescribed light levels without significantly affecting the relative spectral distributions of the LED emissions. Measurements of pupil area (average ± standard deviation) completed after the experiment with a different group of subjects (*n* = 5) were red at 40 lx, 23 ± 11 mm^2^ and blue at 40 lx, 6.6 ± 1.4 mm^2^.

The specific illuminance value of 40 lx was chosen because this value has been shown in previous studies to increase nocturnal alertness after a one-hour exposure [30] and is above threshold and below saturation for melatonin suppression, as described by Rea and colleagues [3]. The light level used in the red light box was selected to have the same photopic illuminance as the blue light, thus providing approximately equal visual stimulation without causing nocturnal melatonin suppression.

### 2.3. Procedures

**Figure 1:**
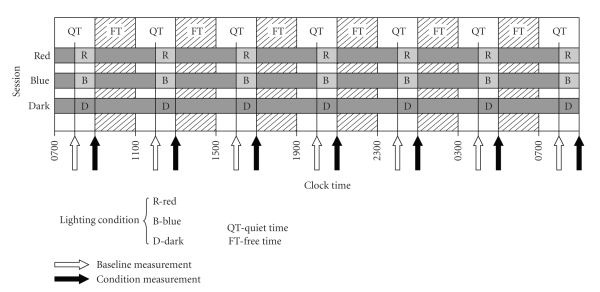
Study protocol. Seven equally spaced baseline measurements were always taken after three hours in the dark (open arrows), except for the first baseline measurement that was taken after one hour in the dark. Subjects were assigned to one of three sessions (red, blue, and dark) corresponding to one of the three lighting conditions they would experience during that session (R, B, and D). Except for the last one, lighting condition measurements were taken one hour after and three hours before each baseline measurements.

Figure 1 shows the course of the 27-hour experiment that employed seven sample times for the *baseline* biomarker measurements (open arrows) and seven sample times for the *lighting condition* measurements (filled arrows). Subjects were assigned to one of three sessions, red, blue, or dark, according to a prescribed schedule designed to counterbalance exposures to the three lighting conditions across subjects. Subjects started each session at 07:00 (always on Fridays) to 10:00 the following day at least one week apart. The experiment ran from March 2008 to March 2009.

One hour prior to making the baseline measurements until completion of the lighting condition measurements (2 hour duration) subjects sat quietly without talking, reading, or otherwise interacting with their environment; these periods were designated quite times (QT), illustrated in Figure 1. All other times were designated as free times (FT) when subjects were given scheduled meals or they could drink noncaffeinated nourishment, talk with each other, or interact with their laptop computers while they sat in the dim (<3 lx of light at the eye) laboratory. If subjects used their computers, they were required to cover the display with an orange filter supplied by an experimenter having a transmittance of less than 2% from 380 to 550 nm. Displays were also dimmed to the minimum brightness.

At all times during a 27-hour session, subjects remained sitting except when they needed to use the restrooms adjacent to the laboratory. They were permitted to use the restroom only during FTs; while absent from the laboratory for this purpose, they were required to wear dark sunglasses enhanced with neutral density filters (less than 5% transmittance). Ten minutes before the end of an FT, subjects were asked to brush their teeth using disposable toothbrushes provided to them while, again, wearing the enhanced sunglasses. They were asked not to use toothpaste so that there would be less chance of saliva sample contamination.

The data collection times for the baseline measurements were at 08:00, 12:00, 16:00, 20:00, 00:00, 04:00, and at 08:00 the following day. Lighting condition measurements were taken approximately one hour after each baseline measurement and always following a one-hour exposure to 40 lx of the blue (B) or to 40 lx of the red (R) lights or while continuing to sit quietly in the dark (D). Experimenters in the room continuously assured that subjects remained awake with their eyes open throughout the entire session, and they were still more carefully monitored during the light exposure periods to ensure compliance with the protocol.

### 2.4. Saliva Collection and Analysis

Saliva samples were collected using the Salivette system from Alpco Diagnostics. This system consists of a centrifuge vessel with a suspended insert in which a cotton swab is placed. To collect the saliva, the cap was removed, and subjects put the tube against their lips and took the cotton swab into their mouths without touching it with their hands. The subjects then chewed the swab to impregnate it with saliva. Between 1-2 ml of saliva was required for the analyses. After chewing the cotton, they then spit the cotton back into the suspended insert, and the cap on the tube was replaced. The vessels containing the suspended saliva-impregnated cotton swabs were then spun in a centrifuge at 1000× g for five minutes, causing the saliva to collect at the bottom of the centrifuge vessel. Saliva samples were frozen for transport to a laboratory for melatonin, cortisol, and alpha amylase assays (Salimetrics, LLC, State College, PA). The sensitivity of the saliva assay for melatonin radioimmunoassay was 0.7 pg/ml, and the intra- and interassay coefficients of variability (CVs) were 12.1% and 13.2%, respectively. The limit of detection for the cortisol assay was 0.0036 *μ*g/dl, and the intra- and interassay CVs were 3.6% and 6.4%, respectively. The limit of detection for the alpha amylase assay was 0.01 u/ml, and the intra- and interassay CVs were 7.2% and 5.8%, respectively.

## 3. Results

The analyses were divided into *baseline* measurements and *lighting condition* measurements. Again, baseline measurements were collected to examine the simultaneous circadian variations in melatonin, cortisol, and alpha amylase production levels for a full day of sleep restriction, and the lighting condition measurements were collected in this context to assess the impact of nighttime and daytime light exposures on these three biomarkers. 

### 3.1. Baseline Measurements

Although complete data for cortisol and for alpha amylase were available from all 12 subjects, complete melatonin data from only seven of these subjects were available for evaluation. Unfortunately, the laboratory that performed the assays reported melatonin levels greater than 50 pg/ml in the saliva samples as simply “greater than 50 pg/ml.” The samples were destroyed by the laboratory after measurement and could not be reassayed. These inexact sample values had to be excluded from the statistical analyses. Consequently, complete melatonin data from just seven subjects (5 males and 2 females) are reported here. Data for cortisol and alpha amylase are for the 12 subjects.

Data from every subject were normalized to the grand mean of each data set, melatonin, cortisol, and alpha amylase, and submitted to three sessions (corresponding to the three lighting conditions D, R, and B) by six measurement times (08:00, 12:00, 16:00, 20:00, 00:00, and 04:00) repeated measures analysis of variance (ANOVA). Data collected during the final measurement time, 08:00 the following day, were not included in the ANOVAs; as discussed below, a subsequent post hoc statistical comparison was made between the three biomarker concentrations obtained during the first and the last sampling periods (i.e., at 08:00). All three ANOVAs supported the same inferences. There was no significant main effect of sessions, and there was no significant interaction between sessions and measurement times. These finding support the inference that the baseline measurements were independent of the lighting condition exposures three hours prior to those baseline measurements and that the data from all three sessions can be combined to better characterize the simultaneous circadian variations in melatonin, cortisol, and alpha amylase. The main effect of measurement times was highly significant (*P* < .0001) from all three ANOVAs, suggesting that each outcome measure followed a circadian pattern over 24 hours.

**Figure 2:**
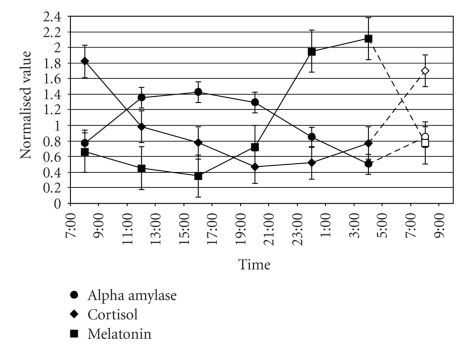
Baseline measurements. Normalized concentration levels of melatonin, cortisol, and alpha amylase (closed symbols) measured under constant dark conditions while subjects were continuously awake for one day together with those collected during the last measurement period (open symbols) at the same clock time as for the first measurement period (08:00).

Again, these findings from the inferential statistics support the conclusion that the baseline measurements for all three sessions were independent of the lighting condition measurements for all three measures. Moreover, since the two lighting conditions (B and R) had no statistically reliable, differential effects on any of the three outcome measures relative to those obtained during the control dark (D) lighting condition, there is no reason to suppose that the phase relationships among these biomarkers were differentially affected by the lighting conditions over the 27-hour course of the study. Therefore, the curves in Figure 2 can be taken to represent, within statistical uncertainty, the baseline rhythms for melatonin, cortisol, and alpha amylase during sleep restriction. 

Melatonin, synthesized by the pineal, follows well-established expectations showing maximum values in the dark at 00:00 and 04:00, with minimum levels from 12:00 to 20:00. Cortisol levels also follow a circadian pattern with peak levels at 08:00 and lowest levels between 20:00 and midnight. Alpha amylase levels, too, follow a clear circadian pattern with peak levels between 12:00 and 16:00 and minimum levels at 04:00. 

The normalized means from the final measurement time, collected at the same clock time as the first measurement time (i.e., 08:00), are also shown in Figure 2. Post hoc statistical comparisons between the first and last times of data collection for each of the three outcome measures showed no statistically significant differences. Over the course of the experiment with subjects staying awake continuously for 27 hours, melatonin, cortisol, and alpha amylase levels returned to, or very near to, the same levels measured at the start of the experiment. This further supports the conclusion, again, that within statistical uncertainty there were no differential effects of the lighting conditions on the phase or amplitude of these three biomarkers and, therefore, the curves in Figure 2 can be taken to represent simultaneous baseline measurements of circadian rhythms for melatonin, cortisol, and alpha amylase during sleep restriction.

### 3.2. Lighting Condition Measurements

**Figure 3:**
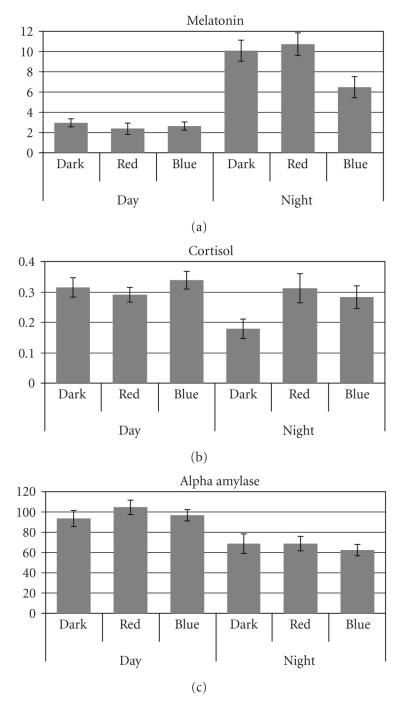
Lighting condition measurements. Mean normalized concentration levels of melatonin (a), cortisol (b), and alpha amylase (c) collected during the day (09:00, 13:00, and 17:00) and during the night (21:00, 01:00, and 05:00) following one hour exposures of 40 lx of narrowband red (R) and blue (B) lights while subjects were continuously awake for one day, together with mean normalized concentration levels collected at the same clock times under constant dark (D) conditions.

In order to determine if light had an acute effect on the outcome measures over the 27-hour protocol, each data set (melatonin, cortisol, and alpha amylase) collected at the end of each lighting condition were submitted to three lighting conditions (D, R, and B) by two times of day (day or night) by three measurement times (first, second, and third measurement during the day and during the night) repeated measures ANOVAs. Again, the three lighting condition measurement times were one hour later and three hours before the baseline measurement times, at 09:00, 13:00, and 17:00 during the day and at 21:00, 01:00, and 05:00 during the night. The times of day factor was utilized in the ANOVAs to determine if there were measurable differences in melatonin, cortisol, and alpha amylase between day and night. Since, however, it was of primary interest to determine if light had differential effects on these outcome measures during the day and during the night, the interaction between lighting conditions and times of day was of more interest than the main effects of either times of day or lighting conditions; the interaction plots for all three outcome measures are shown in Figure 3. Data from the last of the seven measurement periods were not included in these ANOVAs because it was a repetition of the first measurement time and could not be unambiguously categorized as either day or night. 

#### 3.2.1. Melatonin

As previously described, not all of the data could be used for the melatonin analysis. As with the baseline data, complete data from just seven subjects are reported here. This ANOVA revealed a significant main effect of lighting conditions (*F*_2,12_ = 4.3; *P* = .04), a significant main effect of times of day (*F*_1,6_ = 47.2; *P* < .0005), and a significant lighting conditions by times of day interaction (*F*_2,12_ = 5.9; *P* = .02).

As expected, melatonin levels were differentially higher at night than during the day for the three lighting conditions (i.e., there was a significant interaction between lighting conditions and times of day). One-tail, post hoc paired student's *t*-tests showed that melatonin levels were significantly higher at night than during the day for all three lighting conditions (D, R, and B), but at night melatonin levels were significantly lower following the blue light exposure than following both the dark condition (*P* = .006) and following the red light exposure condition (*P* = .004).

#### 3.2.2. Cortisol

Data from twelve subjects were included in the ANOVA for cortisol. There was a significant main effect of measurement times (*F*_2,22_ = 6.4; *P* = .006), a significant lighting conditions by times of day interaction (*F*_2,22_ = 5.5; *P* = .01), and a significant times of day by measurement times interaction (*F*_2,22_ = 38.8; *P* < .001). The main effect of lighting conditions almost reached significance (*F*_2,22_ = 3.1; *P* = .07). 

Based upon the significant lighting conditions by times of day interaction, one-tail, post hoc paired student's *t*-tests were performed. There were no significant differences in cortisol levels between the red and the blue lighting conditions and the dark condition during the day, but cortisol levels were significantly lower following the dark condition than following the blue light (*P* = .001) and the red light exposures (*P* = .004) at night. Furthermore, there were no significant differences between cortisol levels recorded during the day and during the night following both blue light and red light exposures; only following the dark condition was cortisol significantly lower at night than during the day (*P* = .007). These results are consistent with the inference that short-wavelength and long-wavelength lights do not affect cortisol levels during the day relative to darkness and that *both* short-wavelength and long-wavelength lights are capable of bringing nighttime levels of cortisol up to daytime levels following approximately one hour of exposure.

#### 3.2.3. Alpha Amylase

Data from twelve subjects were included in the ANOVA for alpha amylase. A significant times of day main effect (*F*_1,11_ = 12.0; *P* = .005) and a significant times of day by measurement times interaction (*F*_2,22_ = 23.0; *P* < .001) were found, but no significant lighting conditions by times of day interaction was found, as it had been for both melatonin and cortisol. There was, however, a suggestion from the data that light had a differential effect on alpha amylase levels. Using one-tail, post hoc paired student's *t*-tests between alpha amylase levels during the day and during the night for the three lighting conditions showed that the difference between daytime and nighttime alpha amylase levels was larger following the blue light exposure and following the red light exposure than following the dark condition. The difference between daytime alpha amylase levels following blue light exposure was 35 u/ml (*P* < .001) and following red light exposure the difference was 36 u/ml (*P* = .004) whereas the difference between daytime and nighttime amylase levels following the dark condition was only 24 u/ml (*P* = .04). This analysis would suggest then that the two light exposures (blue and red) increased the modulation amplitude of alpha amylase over 24 hours relative to constant darkness.

## 4. Discussion

The baseline measurement curves in Figure 2 show that melatonin, cortisol, and alpha amylase follow circadian patterns, suggesting that the SCN are involved in their daily productions, but the present study shows more clearly that these rhythms have different waveforms and different phase relationships with time of day over the course of the 27-hour study. The results suggest that the HPA and the sympathetic systems buffer one another to maintain homeostasis for an organism [4]; thus, one might expect counter-phased circadian rhythms for cortisol and alpha amylase. However, it is interesting to note that the data in Figure 2 suggest that the two rhythms are not precisely counter-phased. Alpha amylase levels peak in the middle of the day and are lowest during the middle of the night, indicating that the circadian rhythm of this enzyme is more counter-phased with melatonin than with the day-night transitional rhythm of cortisol. Unlike melatonin, however, these results indicate that alpha amylase varies with a symmetric, cosine-like waveform over the 24-hour day, rather than the more binary waveform (high at night and low during the day) rhythm of melatonin.

Also consistent with previous studies these results show that the human circadian system is sensitive to 40 lx of short-wavelength (blue) light at the cornea but is not sensitive to 40 lx of long-wavelength (red) light at the cornea as measured by nocturnal melatonin suppression (Figure 3) [3, 30–32]; obviously too from the literature, blue light-induced melatonin suppression is limited to the nighttime when melatonin levels are high [2]. This replication of many previously published studies of melatonin rhythms and of light-induced melatonin suppression supports the validity of our experimental protocol for assessing baseline rhythms and for evaluating the effects of acute light exposures on cortisol and alpha amylase. 

Of particular interest with respect to exposures to light stimuli at night, this study shows, for the first time, that in contrast to nocturnal melatonin suppression by short-wavelength light alone, *both* short-wavelength and long-wavelength lights affect cortisol levels at night and, although the effects are weaker, both light exposures appeared to affect alpha amylase levels as well. Even though both cortisol and alpha amylase appear to be modulated by both short-wavelength and long-wavelength lights at night, the response characteristics of the systems controlling these two biomarkers are not the same. Cortisol levels are significantly elevated by both the blue and the red lights at night; these same lights appear to have a much diminished effect, if any at all, on cortisol levels during the day. Although only suggestive from the present results, light appears to increase the night-day *contrast *of the rhythmic alpha amylase pattern over the 24-hour day, suggesting a modulation of sympathetic tone by light over the course of the 24-hour cycle [23]. In other words, periodic pulses of light during the day and during the night appear to increase the difference between daytime and nighttime alpha amylase levels. Of significance in this context, since alpha amylase is controlled by the sympathetic system and since the sympathetic system responds quickly to environmental stimuli [20], it would be useful to introduce a higher sampling rate in studies like the present one to better understand the impact of light on the sympathetic system.

The impacts of narrowband (i.e., blue and red lights) light exposures on cortisol and alpha amylase levels in humans have never been reported before. It has been previously shown in rats, however, that polychromatic, white light modulates glucocorticoid production, but only if it is applied at specific circadian times. Buijs and colleagues [17, 33] showed that white light given in the early part of the dark phase (ZT 14) in rats will decrease corticosterone production after 5 minutes of exposure whereas Ishida and colleagues [11] showed that 60-minute exposures to light at a similar circadian time will increase corticosterone production. Both studies showed that light applied later in the subjective night and during the day had no effect on corticosterone production in nocturnal animals. As previously noted, Ishida and colleagues [11] showed that the light-induced corticosterone increase can be exhibited without affecting ACTH production; however, the SCN and sympathetic innervations to the adrenal medulla are necessary for the nighttime, light-induced activation of corticosterone. Given the dual control of glucocorticoid production by the HPA and the sympathetic systems and the temporal dynamics of these two systems, it is reasonable to infer that the daily glucocorticoid rhythm is under control of the HPA system whereas the sympathetic system is responsible for the spike in glucocorticoid levels just prior to activity. Coming back to humans and consistent with this dual control hypothesis, Leproult and colleagues [25] showed that the morning peak in cortisol was enhanced by as much as 50% by bright light exposure (above 2000 lx at the cornea), but this enhancing effect was not seen following application of light in the late afternoon/early evening, when cortisol levels were relatively lower. Scheer and colleagues also showed an effect of morning light exposure, but not evening light exposure, on cortisol levels and on heart rate [24, 26]. The present results extend those from Scheer and colleagues, by showing that light exposure during the middle of the night also increases cortisol production. It should be noted in this context that, like Scheer and colleagues, “evening” light in our experiment (at 21:00) did not show any meaningful light-induced modulation of cortisol. Rather, the significant night-time cortisol response to light was only observed at 01:00 and 05:00. 

Clearly then, the light-sensitive mechanisms affecting nocturnal melatonin suppression are not the same as those affecting cortisol and apparently not the same as those affecting alpha amylase. Although the SCN must be involved in regulating the circadian rhythms of melatonin, cortisol and alpha amylase, as shown here in Figure 2, it is not known whether the photic input acutely modulating these biomarker levels, as shown here in Figure 3, is independent of the SCN or whether the SCN response to light is more complex than presently understood. Of special note, a previous study by Figueiro and colleagues [30] utilizing a different experimental protocol also showed that *both* the blue and the red lights of the same irradiance used in the present study increased nocturnal alertness as measured by electroencephalogram (EEG). The present data, and those by Figueiro and colleagues [30], bring into question then whether the nonvisual pathway mediating nocturnal melatonin suppression is the same as that mediating other nonvisual, photic responses such as the cortisol elevation by light at night observed in the present study. 

In summary, the SCN play a critical role in regulating the endocrine and autonomic nervous systems. Melatonin, cortisol, and alpha amylase each exhibit a robust circadian rhythm under dark conditions across a 24-hour day. These rhythms appear to differ in their temporal dynamics and in terms of their response to environmental light presented at different times during the day and night. Of particular interest with regard to the present study, the photic inputs to the pineal and to the adrenal glands are clearly different; synthesis of melatonin by the pineal gland is only affected by short-wavelength light whereas cortisol production by the adrenal gland is modulated by both short- and long-wavelength light. It would seem too that the sympathetic system response, as measured in terms of alpha amylase concentration, also has a spectral sensitivity to light broader than that leading to the pineal gland response. It remains to be determined whether the suggested broader spectral sensitivity of the alpha amylase response is the same as that leading to the light-induced cortisol response by the adrenal gland. Nevertheless, the present results clearly demonstrate that a photic pathway to the endocrine and autonomic nervous systems exists other than that responsible for nocturnal melatonin suppression.

## References

[1] Refinetti R (2006). *Circadian Physiology*.

[2] Arendt J (1995). *Melatonin and the Mammalian Pineal Gland*.

[3] Rea MS, Figueiro MG, Bullough JD, Bierman A (2005). A model of phototransduction by the human circadian system. *Brain Research Reviews*.

[4] Goldstein DS (2003). Catecholamines and stress. *Endocrine Regulations*.

[5] Miller GE, Chen E, Zhou ES (2007). If it goes up, must it come down? Chronic stress and the hypothalamic-pituitary-adrenocortical axis in humans. *Psychological Bulletin*.

[6] Nicolson NA, Luecken LJ, Gallo LC (2008). Measurement of cortisol. *Handbook of Physiological Research Methods in Health Physiology*.

[7] Halberg F, Barnum CP, Silber RH, Bittner JJ (1958). 24-hour rhythms at several levels of integration in mice on different lighting regimens. *Proceedings of the Society for Experimental Biology and Medicine*.

[8] Guillemin R, Fortier C, Lipscomb HS (1959). Comparison of in vitro and in vivo assaying procedures for rat adenohypophysial corticotropin. *Endocrinology*.

[9] McCarthy JL, Corley RC, Zarrow MX (1960). Diurnal rhythm in plasma corticosterone and lack of diurnal rhythm in plasma compound F-like material in the rat. *Proceedings of the Society for Experimental Biology and Medicine*.

[10] Kalsbeek A, Drijfhout W-J, Westerink BHC (1996). GABA receptors in the region of the dorsomedial hypothalamus of rats are implicated in the control of melatonin and corticosterone release. *Neuroendocrinology*.

[11] Ishida A, Mutoh T, Ueyama T (2005). Light activates the adrenal gland: timing of gene expression and glucocorticoid release. *Cell Metabolism*.

[12] Schibler U, Brown SA (2005). Enlightening the adrenal gland. *Cell Metabolism*.

[13] Kaneko M, Hiroshige T, Shinsako J, Dallman MF (1980). Diurnal changes in amplification of hormone rhythms in the adrenocortical system. *American Journal of Physiology*.

[14] Haus E (2007). Chronobiology in the endocrine system. *Advanced Drug Delivery Reviews*.

[15] Kudielka BM, Buchtal J, Uhde A, Wüst S (2007). Circadian cortisol profiles and psychological self-reports in shift workers with and without recent change in the shift rotation system. *Biological Psychology*.

[16] Szafarczyk A, Ixart G, Alonso G, Malaval F, Nouguier-Soulé J, Assenmacher I (1983). CNS control of the circadian adrenocortical rhythm. *Journal of Steroid Biochemistry*.

[17] Buijs RM, Wortel J, Van Heerikhuize JJ (1999). Anatomical and functional demonstration of a multisynaptic suprachiasmatic nucleus adrenal (cortex) pathway. *European Journal of Neuroscience*.

[18] Oster H, Damerow S, Kiessling S (2006). The circadian rhythm of glucocorticoids is regulated by a gating mechanism residing in the adrenal cortical clock. *Cell Metabolism*.

[19] Rohleder N, Nater UM, Wolf JM, Ehlert U, Kirschbaum C (2004). Psychosocial stress-induced activation of salivary alpha-amylase: an indicator of sympathetic activity?. *Annals of the New York Academy of Sciences*.

[20] Granger DA, Kivlighan KT, El-Sheikh M, Gordis EB, Stroud LR (2007). Salivy *α*-amylase in biobehavioral research: recent developments and applications. *Annals of the New York Academy of Sciences*.

[21] Nater UM, Rohleder N (2009). Salivary alpha-amylase as a non-invasive biomarker for the sympathetic nervous system: current state of research. *Psychoneuroendocrinology*.

[22] Rohleder N, Nater UM (2009). Determinants of salivary *α*-amylase in humans and methodological considerations. *Psychoneuroendocrinology*.

[23] Nater UM, Rohleder N, Schlotz W, Ehlert U, Kirschbaum C (2007). Determinants of the diurnal course of salivary alpha-amylase. *Psychoneuroendocrinology*.

[24] Scheer FAJL, Buijs RM (1999). Light affects morning salivary cortisol in humans. *Journal of Clinical Endocrinology and Metabolism*.

[25] Leproult R, Colecchia EF, L’Hermite-Balériaux M, Van Cauter E (2001). Transition from dim to bright light in the morning induces an immediate elevation of cortisol levels. *Journal of Clinical Endocrinology and Metabolism*.

[26] Scheer FAJL, Van Doornen LJP, Buijs RM (2004). Light and diurnal cycle affect autonomic cardiac balance in human; possible role for the biological clock. *Autonomic Neuroscience: Basic & Clinical*.

[27] Burgess HJ, Fogg LF (2008). Individual differences in the amount and timing of salivary melatonin secretion. *PLoS ONE*.

[28] Strahler J, Mueller A, Rosenloecher F, Kirschbaum C, Rohleder N (2010). Salivary *α*-amylase stress reactivity across different age groups. *Psychophysiology*.

[29] Roenneberg T, Wirz-Justice A, Merrow M (2003). Life between clocks: daily temporal patterns of human chronotypes. *Journal of Biological Rhythms*.

[30] Figueiro MG, Bierman A, Plitnick B, Rea MS (2009). Preliminary evidence that both blue and red light can induce alertness at night. *BMC Neuroscience*.

[31] Thapan K, Arendt J, Skene DJ (2001). An action spectrum for melatonin suppression: evidence for a novel non-rod, non-cone photoreceptor system in humans. *Journal of Physiology*.

[32] Brainard GC, Hanifin JR, Greeson JM (2001). Action spectrum for melatonin regulation in humans: evidence for a novel circadian photoreceptor. *Journal of Neuroscience*.

[33] Buijs RM, Wortel J, Van Heerikhuize JJ, Kalsbeek A (1997). Novel environment induced inhibition of corticosterone secretion: physiological evidence for a suprachiasmatic nucleus mediated neuronal hypothalamo-adrenal cortex pathway. *Brain Research*.

